# Recent Progress and Opportunities for Nucleic Acid Aptamers

**DOI:** 10.3390/life11030193

**Published:** 2021-02-28

**Authors:** Jonghoe Byun

**Affiliations:** Department of Molecular Biology, College of Science & Technology, Institute of Nanosensor and Biotechnology, Dankook University, Dandaero 119, Dongnam-gu, Cheonan-si, Chungnam 31116, Korea; jonghoe@dankook.ac.kr

**Keywords:** aptamer, SELEX, antibody, therapeutics, diagnostics, targeting, COVID-19

## Abstract

Coined three decades ago, the term aptamer and directed evolution have now reached their maturity. The concept that nucleic acid could modulate the activity of target protein as ligand emerged from basic science studies of viruses. Aptamers are short nucleic acid sequences capable of specific, high-affinity molecular binding, which allow for therapeutic and diagnostic applications. Compared to traditional antibodies, aptamers have several advantages, including small size, flexible structure, good biocompatibility, and low immunogenicity. In vitro selection method is used to isolate aptamers that are specific for a desired target from a randomized oligonucleotide library. The first aptamer drug, Macugen, was approved by FDA in 2004, which was accompanied by many studies and clinical investigations on various targets and diseases. Despite much promise, most aptamers have failed to meet the requisite safety and efficacy standards in human clinical trials. Amid these setbacks, the emergence of novel technologies and recent advances in aptamer and systematic evolution of ligands by exponential enrichment (SELEX) design are fueling hope in this field. The unique properties of aptamer are gaining renewed interest in an era of COVID-19. The binding performance of an aptamer and reproducibility are still the key issues in tackling current hurdles in clinical translation. A thorough analysis of the aptamer binding under varying conditions and the conformational dynamics is warranted. Here, the challenges and opportunities of aptamers are reviewed with recent progress.

## 1. Introduction

Aptamers are small oligonucleic acids or peptide molecules that bind to their target with high specificity. Aptamers can be classified as nucleic acid aptamers and peptide aptamers, depending on the random sequence pools used to generate them. In general, however, aptamer refers to nucleic acid aptamers unless clearly indicated. Three decades ago, a revolutionary in vitro selection method for synthetic RNAs that could bind specifically to their target molecules was simultaneously developed by three independent groups [1,2,3]. The structured RNA motifs were termed aptamers based on the Latin words ”aptus” (fitting) or “aptare” (to fit) and the Greek word ”meros” meaning part. High-affinity nucleic acid ligands for a protein were isolated by systematic evolution of ligands by exponential enrichment (SELEX) that depends on alternate cycles of ligand selection from pools of variant sequences and amplification of the bound species [2,3,4,5,6,7]. Several rounds of selection exponentially enriched the population for highest affinity species that can be clonally isolated and characterized. In an earlier stage of aptamer development, RNA libraries were widely used because RNA can easily form secondary structures and fold into three-dimensional structures that have high affinity to their respective targets. The advances in generation of aptamers, application in biosensing, biotechnology and medicine, and the limitations and future directions of aptamers in target specific delivery and real-time detection have been recently reviewed [8,9]. Here, the challenges and opportunities of aptamers are discussed with recent progress.

## 2. Nucleic Acid Aptamer

Aptamers are short, single-stranded RNA (ssRNA) or DNA (ssDNA) molecules that assume various shapes, owing to their tendency to form helices or single-stranded loops. These small nucleic acid ligands, often referred to as chemical antibodies, enable recognition and binding to their targets with high specificity and affinity, often surpassing those of antibodies. The dissociation constants of aptamers are usually in the pico- to nanomolar range. The binding of an aptamer to a target molecule is achieved by its own special three-dimensional structure, with features such as hairpins, internal loops, bulges, pseudoknots, and G tetramers [10]. The structural interaction between the aptamer and its target is similar to that of an antigen–antibody reaction. The binding is mediated via van der Waals forces, hydrogen bonding, electrostatic interactions, stacking of flat moieties, and shape complementarity [11].

Aptamers are capable of wrapping around a small molecule target or fitting into clefts and gaps within the surface of much larger target molecules. This flexible nature of aptamers, to fold into or around the complex surfaces of a target molecule, means that aptamers can select a wide range of targets, including traditionally difficult targets for other affinity reagents. Indeed, the targets of aptamer may include, but are not limited to peptides, proteins, metabolites, small organic compounds, carbohydrates, biological cofactors, metal ions, toxins, and whole organisms such as virus, pathogenic bacteria and yeast, and mammalian cells [12,13,14,15,16,17,18,19,20,21,22,23,24].

## 3. Systematic Evolution of Ligands by Exponential Enrichment (SELEX)

**Figure 1:**
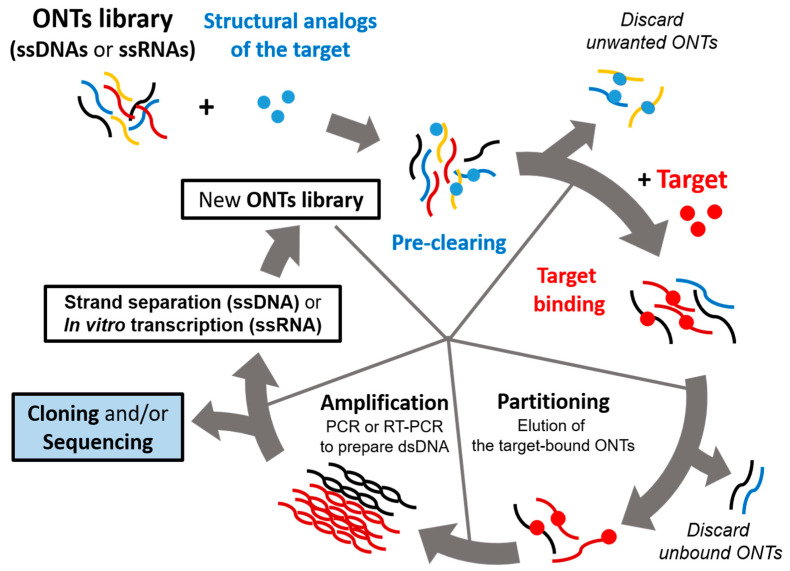
Schematic representation of a systematic evolution of ligands by exponential enrichment (SELEX) procedure. The iterative in vitro selection procedure known as SELEX is used to obtain oligonucleotide ligands that bind to their target with high affinity and specificity. SELEX starts with oligonucleotide (ONT) library consisting of single-stranded DNA or RNA molecules (ssDNAs or ssRNAs). An initial pool of 10^14^–10^15^ random oligonucleotide (ONT) strands are subjected to binding with the target. Unbound ONTs are discarded, and RT-PCR or PCR is performed to amplify the target-bound ONTs. This selection process is repeated 4–20 times depending on its particular case using amplified ONTs as a new pool, which should be enriched with target-specific binders. Negative selection (preclearing) can be included in either RNA or DNA SELEX protocols, which is achieved by passing the nucleic acid pool over a supporting matrix in the absence of the target. This step aims at eliminating the oligonucleotides that bind the matrix in a target-independent manner.

Aptamers are screened by a repeated cycle of binding, washing, purification, and amplification steps (Figure 1). These in vitro selection rounds are iterated with exponential amplification of the bound oligonucleotides by PCR for DNA libraries or RT-PCR for RNA libraries. In a typical SELEX experiment, the nucleic acid library, which consists of 10^14^–10^15^ random oligonucleotide sequences, is incubated with a target molecule [8,12]. These sequences in the pool have unique three-dimensional structures defined by the combination of interactions that include base pairing, stacking, sugar packing, and noncanonical intramolecular interactions [25]. This structural complexity in the pool establishes a high probability of selecting an oligonucleotide that can interact avidly and specifically with the target of interest [26]. Then, the target-bound oligonucleotides are separated from the unbound strands. The binding forces mediating the aptamer–target interactions may include hydrogen bonding, electrostatic interaction, salt bridges, hydrophobic effect, π-π stacking, and van der Waals forces [27]. The target-bound DNA or RNAs are eluted from the target molecule and amplified via polymerase chain reaction to seed a new pool of nucleic acids. This selection process is repeated for several rounds (between four and 20) with increasingly stringent conditions, which ensure that the nucleic acids obtained have the highest affinity to the target molecule [28]. 

Two alternative protocols can be followed to complete the SELEX. In the traditional SELEX, the selected oligonucleotides from the final round are cloned and sequenced for aptamer identification. Alternatively, deep sequencing of the selection pools after each cycle can be performed [28]. This high-throughput SELEX (HT-SELEX) allows for understanding of the selection process and the relationships between sequences in different selection rounds. Comparative sequence analysis can identify consensus sites that are potentially involved in target recognition. Moreover, HT-SELEX can reveal previously not appreciated properties of the SELEX protocol. Indeed, examination of earlier pools using HT-SELEX provides an opportunity to uncover potential binders that might otherwise have been lost in later steps of the selection process. More importantly, by analyzing the relative changes of consecutive selection rounds with respect to the properties such as sequence diversity and mutation rates, the method provides an unprecedented opportunity to gain deeper insights into the selection process itself [29,30,31]. Tracing the progress of the selection process and the rational feedback with desired properties would make the SELEX protocol more rapid and efficient. To further optimize the SELEX procedure, other techniques such as surface plasmon resonance (SPR) and capillary electrophoresis (CE) can be integrated, which would help to increase biostability and improve binding performances.

Selection of aptamers using the original SELEX, which is often cumbersome and time-consuming, provides aptamers with a limited number of functional groups, namely four bases of DNA or RNA. However, newer SELEX technology such as X-aptamers can increase diversity greatly because it provides an unlimited number of functional groups [32]. In X-aptamers, a bead-based selection method is used for random incorporation of drug-like moieties onto next-generation aptamers for enhanced binding [33].

Recently, a novel approach for developing aptamers against membrane proteins was reported [34]. By using surrogate virus-based SELEX called viro-SELEX, multiple advances could be incorporated into the flu diagnostics, including highly sensitive, specific, portable, rapid, and quantitative point-of-care testing diagnostic tools for the future. In viro-SELEX, surrogate viruses that contained target proteins on the surface of an enveloped virus (baculovirus) were used instead of purified proteins. Through this novel approach, a pair of aptamers that specifically interact with the hemagglutinin protein of influenza virus subtype H3N2 were screened. This aptamer pair was successfully incorporated into a very sensitive point-of-care diagnostic system employing lateral flow assay [35]. This success bodes well for the novel theragnostic system for other viruses including SARS-COV-2.

An improved aptamer can also be developed by computational methods to target different structural proteins of SARS-CoV-2 for diagnosis and therapeutic purposes. Recently, great strides have been made in artificial intelligence and this technology has been integrated into aptamer discovery, combined with in silico methods and bioinformatic tools [29,30]. These computational methods are expected to reduce the time and cost associated with aptamer selection and help the drug development process. For example, in silico methods are useful for ranking the candidate aptamers from HT-SELEX data, clustering a huge number of aptamer sequences, and finding motifs amidst a set of significant nucleic acid aptamers [36]. Although high-throughput sequencing (HTS) enables a broader and more accurate analysis of the obtained aptamers, giving information on their abundance along with the identification of functional and rare motifs, the vast amount of data generated requires more sophisticated bioinformatic tools to identify the best candidates among the hundreds of millions of sequences obtained. Further optimization steps such as shortening and modifying the sequences of candidate aptamers can also benefit from computational methods. In addition, a decreased amplification bias, nonspecific adsorption, and expansion of different base types can be expected [37].

## 4. Advantages of Aptamers

Aptamers have several advantages over commonly used small-molecule ligands. The primary advantages of aptamers include good stability (high thermal stability), tailored specificity and affinity with almost any target, minimal toxicity, and scalability of production (Table 1). Aptamers also offer advantages over antibodies as they can be engineered completely in a test tube, are readily produced by chemical synthesis, possess desirable storage properties, and elicit little immunogenicity in therapeutic applications [8,31]. Since they are chemically synthesized, batch-to-batch variation is eliminated and less time is required for development. Moreover, they have excellent tissue permeability, unlimited availability of targets including nonimmunogenic epitopes, and modifiability [38]. These numerous advantages offered by aptamers have led to many applications across a diverse range of diagnostics and therapeutics.

## 5. Aptamers as Therapeutic Agents

**Figure 2:**
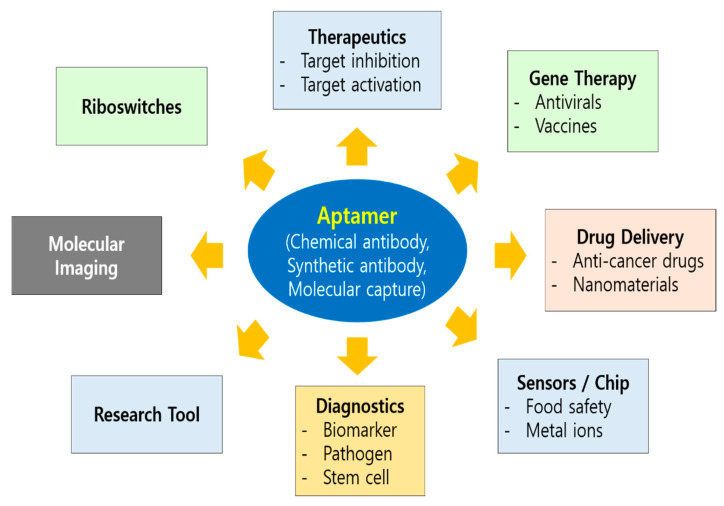
Diverse areas of aptamer application. Nucleic acid aptamers can be applied to various disciplines of biomedical arena such as theragnostics, molecular imaging, drug delivery, biosensors/chip, and gene therapy which includes antivirals and vaccines. As an invaluable research tool, aptamer also foster basic studies including riboswitches in nature. Therapeutic application of aptamer can be categorized as antagonists (target inhibition), agonists (target activation), delivery vehicles, and gene therapy.

Use of aptamers for therapeutic purposes began in 1990 when Tuerk and Gold performed the first SELEX experiment for the selection of an RNA aptamer against bacteriophage T4 DNA polymerase [2]. Sullenger et al. reported the inhibitory effect of TAR-containing sequences (TAR decoys) on an HIV-1 viral infection in host cells. These TAR decoys (aptamers) prevent the Tat protein from binding the endogenous TAR RNA, thereby inhibiting HIV gene expression and replication. TAR decoy RNA-mediated HIV inhibition was also suggested to be effective against natural HIV isolates in spite of their hypervariable nature, because replication of SIVmac was also inhibited in cells expressing HIV-1 TAR decoys [39]. Since then, a number of aptamers have been studied in preclinical and clinical development with different strategies employed for the therapeutic application of aptamers, which includes aptamers as antagonists or agonists, and delivery vehicles (Figure 2). Most aptamers that have been developed so far as therapeutics can be categorized as antagonist, which inhibits the target. All aptamers that have entered clinical trials so far act as antagonists [40]. An opportunity to improve the design and therapeutic efficacy of the antagonistic aptamer would help meet the expectations of the clinic.

Current efforts to combine different aptamer functionalities with other molecules of interest such as reporter groups, cholesterol, nanoparticles, chemotherapeutic agents, siRNA, and antisense oligonucleotides should provide a wide range of applications of multivalent aptamers [41,42]. Since a broad spectrum of chemical approaches is available to conjugate aptamer modules with each other and with other molecules of interest, nucleic acid aptamers provide a much more convenient platform for design of multivalent aptamers [42,43]. On the one hand, similar to bispecific therapeutic antibodies called diabodies [44,45], by improving characteristics such as great tolerance to chemical modifications and high stability, a set of multivalent aptamers can be newly designed for broader applications in the near future. On the other hand, only a small number of aptamers have been developed as agonists [40]. RNA aptamers were developed for targets such as HER3/ERBB3, OX40, 4-1BB, CD40, and CD28 [46,47,48,49,50], whereas DNA aptamers were screened for targets like human VEGFR-2 and insulin receptor [51,52]. In the latter case, the agonistic aptamer named IR-A48 specifically bound to insulin receptor, but not to IGF-1 receptor, showing biased activity toward subsets of the insulin signaling pathway, and hence the possibility for developing allosteric-biased agonists to a specific membrane receptor through SELEX [52]. Other therapeutic targets of aptamer studied so far include thrombin, nucleolin, and prostate-specific membrane antigen [43,53,54]. In addition, aptamers have been used to treat aging-related disorders [55], obesity and diabetes mellitus [56,57], cardiovascular diseases [58], infectious diseases [59,60], blood coagulation [61], bone diseases [62], immunological diseases [63], and cancers [64]. 

In 2004, the first aptamer therapeutic, Macugen (Pegaptanib), was approved by FDA. Other aptamers are currently being evaluated in clinical trials [65]. To date, nine RNA and five DNA aptamers have undergone clinical trials for the treatment of various conditions such as macular degeneration, coagulation, oncology, and inflammation (Table 2). The detailed indications include choroidal neovascularization, intravascular thrombus, acute coronary syndrome/coronary artery disease, von Willebrand factor related disorders, von Hippel–Lindau syndrome (VHL), angiomas, acute myeloid leukemia, renal cell carcinoma, non-small cell lung cancer, and thrombotic thrombocytopenic purpura.

Pegaptanib is a pegylated RNA aptamer of twenty-eight nucleotides that terminates in a pentylamino linker, to which two 20 kD monomethoxy polyethylene glycol (PEG) units are covalently attached via the two amino groups on a lysine residue [66]. Pegaptanib specifically binds to isoform 165 of vascular endothelial growth factor (VEGF165), thereby inhibiting VEGF165 binding to its VEGF receptors. This antagonistic action of Macugen prevents abnormal angiogenesis, vascular permeability, intraocular hemorrhage, and inflammation, all of which are thought to contribute to the progression of the neovascular (wet) form of age-related macular degeneration (AMD), a leading cause of blindness over the age of 50 [67,68]. The first annual sales of Macugen in 2005 exceeded 200 million USD. This initial success of Macugen, however, has met stiff competition from Lucentis (ranibizumab, Novartis), which appears to be more effective than Macugen. Competition also came from the off-label use of the anti-VEGF mAb bevacizumab (Avastin, Genentech), which is less expensive. Macugen now retains a relatively small and precarious market share for the treatment of AMD [69].

Aptamer-based targeting is an attractive modality in molecular medicine (Figure 2). Aptamers against cell surface receptors that are internalized have been exploited to deliver a variety of cargoes into cells [70]. As targeting ligands in drug delivery systems, aptamers can be used to deliver molecules that are not otherwise taken up efficiently by cells or to limit delivery of molecules that are efficiently taken up by cells that express aptamer targets. Here, aptamers function to guide the systems to the desired cells and tissues to maximize treatment efficacy and minimize systemic toxicity [71]. The aptamers can be internalized either by macropinocytosis or receptor mediated endocytosis [72]. The perfect target for aptamer-mediated delivery would be the one that is highly expressed on target cells only, which in turn should lead to efficient internalization. Although a perfect target may be hard to find, a proof-of-concept study has shown that aptamers can mediate cell type-specific delivery [73]. Cargoes that can be used are enzymes, toxins, chemotherapeutic agents, imaging agents, miRNAs, and siRNAs [74]. 

## 6. Aptamers in Diagnostics

The increasing need for precise quantification, unbiased measurement, and intellectual separations have ushered in several innovations in diagnostics. One of those innovations is nucleotide aptamers that can mimic and replace antibodies in a wide variety of applications, including lateral flow devices, ELISA, and affinity chromatography. Compared with antibody, the aptamers selected by in vitro selection have merits such as tenacity, conformational flexibility, target affinity, and analytical selectivity (Table 1). These characteristics of aptamers are suited for point-of-care device and field-based testing kits which employ gold nanoparticles and quantum dots for readouts. For diagnostic application, aptamers first must be immobilized to a surface where the requirements of the desired analytical methods determine the nature of substrate and chemistry for aptamer immobilization (Table 3). 

Aptamers are employed in biosensing devices as probes. The specificity of aptamers as molecular recognition element makes aptamer-based sensors (aptasensors) uniquely suited for selective detection of targets in blood, food samples, or pathogens [75]. They are superior to conventional biosensors that use antibodies as they are stable, have high affinity to targets, modifiable, and can be developed for a wide range of targets, using various transduction mechanisms. One of the most common aptasensor formats is the duplexed aptamer (DA) [76]. DAs are aptasensors that contain two nucleic acid elements coupled via Watson–Crick base pairing. One is an aptamer sequence, which serves as a ligand-specific receptor, and the other is an aptamer-complementary element (ACE), such as a short DNA oligonucleotide, which is designed to hybridize to the aptamer. The ACE competes with ligand binding, such that DAs generate a signal upon ligand-dependent ACE-aptamer dehybridization. DAs possess intrinsic advantage such as the design that can be generalized across DNA and RNA aptamers, DAs are compatible with many readout methods, and DAs are inherently tunable on the basis of nucleic acid hybridization. Owing to their tractability, versatility and ease of engineering, DA biosensors bear a great potential for the development of new applications and technologies in fields ranging from analytical chemistry and mechanistic modeling to medicine and synthetic biology [76].

Aptasensors can be classified as electrochemical, optical, or field-effect transistor (FET)-based methods depending on the type of transduction mechanisms employed [84]. Electrochemical impedance spectroscopy (EIS) is a widely used tool for unraveling complex nonlinear processes. This versatile nondestructive steady-state technique is used to detect the dynamics of biomolecular interactions in the field of electrochemical sensors [80]. Performed in the presence of a redox agent, EIS is an ideal tool to measure the molecular interactions of electrochemically inactive compounds taking place on the electrode surface [85]. In particular, EIS-based biosensors are well-suited to the detection of binding events occurring on the transducer surface since minute changes in analytes can be easily and rapidly detected on a biosensor surface [85].

Compared to other molecule-based assays or diagnostic technology, aptasensors are simple, rapid, less expensive, and can be used in real time. In addition, there is less contamination and no need for sample preparation. Similar to molecular diagnostics, aptasensors can be incorporated into multiplex systems with sensitivity and specificity. The use of aptamers in diagnostics has fewer limitations related to health issues than the aptamers used for therapeutic purposes.

Despite these apparent advantages, the main obstacle of aptamer in diagnostics is related to the lack of standardized protocols. The different aptamers generated in the same laboratory against the same target often differ in their primary structures, affinities and specificities, and other chemical parameters. As a consequence, the protocol developed for one aptamer cannot be applied for other aptamers [86]. The aptamer-based diagnostics has to be reproducible and predictable based on the accomplishments made so far in understanding the interactions between a variety of substrates and aptamers and how these interactions can be harnessed as colorimetric or other reporters in biosensors; however, the paucity of well-characterized aptamers for specific targets is still a limiting factor. For the diagnosis of human diseases, standardized kits and protocols based on well-characterized aptamers with optimum characteristics must be developed together with generation of solid databases for the characterized aptamers.

Recently, to overcome some of the challenges faced by conventional protein imprinting, a synthetic receptor sensor based on biomolecular recognition elements and molecular imprinting has been developed [87]. In this study, the molecularly imprinted polymer (MIP) cavity was created by complexing a thiolated DNA aptamer with prostate-specific antigen (PSA) followed by immobilization on the surface of a gold electrode and controlled electropolymerization of dopamine around the complex, which served to entrap the complex and to localize the PSA binding sites at the sensor surface, holding the aptamer in, or near to, it′s binding conformation. This MIP cavity would act synergistically with the embedded aptamer to form a hybrid receptor (apta-MIP), displaying recognition properties superior to that of aptamer alone. Employing electrochemical impedance spectroscopy (EIS), the apta-MIP sensor showed high sensitivity with a linear response from 100 pg/mL to 100 ng/mL of PSA and a limit of detection (LOD) of 1 pg/mL, which was three-fold higher than aptamer alone sensor. Furthermore, the sensor demonstrated low cross-reactivity with a homologous protein (human Kallikrein 2) and low response to non-specific serum proteins [87]. In a similar study, a signal-on built-in marker electrochemical aptasensor was fabricated for the detection of human PSA. This aptasensor detected PSA with a linear concentration range of 125 pg/mL to 128 ng/mL and a LOD of 50 pg/mL, which was successfully applied to detect PSA in the blood serum samples [88]. Very recently, an impedimetric aptasensor for the detection of HER2 breast cancer biomarker was developed and optimized. Two architectures were used to immobilize DNA aptamers on gold screen-printed electrodes. The first platform was composed of self-assembled monolayer (SAM) made from a mixture of thiolated DNA aptamers specific for HER2 and 1-mercapto-6-hexanol (MCH), while the second one was a ternary SAM composed of the same aptamer and 1,6-hexanethiol (HDT). Both platforms were further passivated with MCH and blocked with bovine serum albumin. EIS provided 172 pg/mL and 179 pg/mL as LODs in a dynamic range from 1 pg/mL to 100 ng/mL. Electrochemical aptasensors are highly promising for medical diagnostics and ternary layers could improve the LOD by reducing non-specific interactions [89].

## 7. Hurdles and Opportunities

Despite much initial expectations, many aptamers, so far, have failed to meet the requisite safety and efficacy standards in human clinical trials. Although several aptamers are still in human clinical trials (Table 2), there seems to be a long way to go for these aptamers. The pharmacokinetic properties of aptamers are also relatively difficult to control, as they can degrade, be excreted, or be involved in metabolic processes at different rates throughout the body. These factors could strongly affect the duration of aptamer efficacy, which is of critical importance in a clinical setting. Most common hurdles and their solutions are summarized in Table 4.

Aptamers are susceptible to nucleases that are present in many biological samples. This is particularly true for RNA, having the 2′OH group, which can electrophilically attack the phosphate of the nucleic acid backbone. To resist nuclease-mediated degradation, protective groups can be introduced at the 2-position of ribose sugar [12]. Consistent with these approaches, Pegatinib is PEGylated (a 40 kDa polyethylene glycol substituent is linked to the 5′ molecular terminus) and contains a phosphorothioate 3′-3′ deoxythymidine cap to promote nuclease stability. All of the purine ribose sugars are 2′-O-methylated and the pyrimidine ribose sugars all 2′-fluorinated [65,66].

Aptamers mostly target molecules on the surface of cells or are present in the blood stream; however, they have difficulty entering cells and finding intracellular targets without the aid of a cell-penetrating component. One way to address this issue is to use recombinant vectors that enter cells, and then express the aptamer within the cell, which could overcome the problem of cell penetration. Recently, intracellular applications of aptamer were investigated based on the superior target recognition of aptamers [90]. The aptamers with better structure recognition on the intracellular epitopes have quickly emerged as intracellular targeting agents and intracellular imaging tools. Intracellular aptamers, or intramers have versatile functions ranging from intracellular RNA imaging, gene regulation, and therapeutics to allosteric modulation. The first fluorogenic RNA aptamer ”Spinach” was capable of turning on the fluorescence upon binding of small-molecule fluorophores, 3,5-difluoro-4-hydroxybenzylidene imidazolinone (DFHBI), mimicking the green fluorescent protein (GFP) [91].

The environment during aptamer selection must also be carefully considered in detail [25,31,92,93,94]. Aptamers are usually created using target molecules of high purity. Aptamers that are screened under this condition do not guarantee their performance in the natural environment of the cell where multiple components are present. The structure, function, binding affinity, and specificity of aptamers can be changed in clinical samples. Since aptamer selection directly depends on the environment during SELEX, the components of the operating buffer system such as the specific ions, ionic strength, and pH can dictate the predominant structures of oligonucleotides in the pool. The negative charge distribution of nucleic acid at physiological pH and electrostatic interactions along the strands within the particular environment can directly affect the three-dimensional structure of nucleic acids, and thus the target binding. Structures of short oligonucleotides-like aptamers (20~100 nt) are also affected by the incubation temperature and duration. Moreover, RNA aptamers are more prone to hydrolysis in alkaline pH and high temperatures. The susceptibility to hydrolysis may be circumvented by using chemically substituted nucleotide analogs during SELEX [95,96]. However, aptamer selection failure can be common due to significant uncertainties in PCR bias, PCR artifacts, and background binders [97]. To optimize the selection process and aptamer properties for enhanced affinity and specificity, researchers have developed improved strategies, such as SOMAmers (slow off-rate modified aptamers) and Spiegelmers, bead-based selection, CE-SELEX, microscopic SELEX, Cell-SELEX, in vivo SELEX, capture-SELEX, and microfluidics technology [98]. SOMAmers are ssDNA aptamers that increase chemical complexity of aptamers by using base-modified dUTP and 5-methyl-dCTP [99]. Spiegelmers are mirror-image RNA aptamers (L-form) that are resistant to endonuclease [100]. 

Although aptamers are promising ligands for targeted theragnostics, one potential barrier for translation into clinic is the possible loss of targeting efficacy in the human body. Indeed, the rapid clearance of nanoparticles due to the immune response to the nanoparticle, aggregation of small nanoparticles, protein corona, and enzymatic cleavage of the aptamer can be the mechanism underlying the loss of aptamer-based targeting in vivo [101]. Improvement in the smart design coupled with deeper knowledge of the human body should provide solutions to this problem in the near future.

## 8. Xeno-Nucleic Acids

Xeno-nucleic acids or XNAs are synthetic genetic polymers whose backbone structures are distinct (”xeno” means ”foreign, strange”) from those found in nature. In XNA, the natural sugar found in DNA and RNA has been replaced with a different type of sugar moiety, thus, conferring resistance to nucleases. XNAs have attracted significant attention as new polymers for synthetic biology and molecular medicine because of their unique physicochemical properties such as increased biological stability, enhanced chemical stability, altered helical geometry, or even elevated thermodynamics of Watson–Crick base pairing [102].

One of the representative XNAs is α-L-threofuranosyl nucleic acid or threose nucleic acid (TNA) which is under active investigation as a source of nuclease resistant aptamers with practical application in theragnostics. For example, TNA aptamers were selected in vitro to bind PD-L1 protein and inhibit its interaction with PD-1. These biologically stable TNA aptamers not only bound target proteins with nanomolar affinities but also effectively blocked PD-1–PD-L1 interaction in vitro. In a mouse xenograft model of colon cancer, the injected TNA aptamer N5 was found to be specifically accumulated at the tumor site and significantly inhibited tumor growth in vivo [103]. However, nearly all of the XNA aptamers produced thus far have been generated against protein targets, raising doubts about the ability of XNA aptamers to recognize small molecule targets. However, recent a study has shown that an XNA aptamer that has affinity to a small-molecule target such as ochratoxin A (OTA) can be obtained [104]. Selection experiments against OTA yielded aptamers having affinities in the mid-nanomolar range, with the best binders possessing KD values comparable to or better than those of the best previously reported DNA aptamer to OTA. Moreover, the TNA could be incubated in 50% human blood serum for seven days and retain binding to OTA with only a minor change in affinity, whereas the DNA aptamer was completely degraded. This demonstrates the remarkable biostability of the TNA aptamer and the high level of selectivity in a large background of competing biomolecules. More recently, an ATP-binding aptamer composed entirely of TNA was obtained which showed high affinity to ATP and strong specificity against other naturally occurring ribonucleotide triphosphates [105]. TNA aptamer sequence can be minimized to a length that is compatible with chemical synthesis, which enables new applications that were previously not feasible for XNA aptamers due to the limited practical scale of enzymatic synthesis [104]. 

The search for nucleic acid analogs with even simpler backbones has led to studies of the glycerol nucleic acids (GNAs), which have a three-carbon, acyclic backbone [106]. The S isomer of GNA can form stable antiparallel duplexes with itself and RNA, Bst DNA polymerase was found to catalyze full-length DNA synthesis on a dodecamer GNA template and certain thermophilic and mutant polymerases have been developed for incorporation of modified nucleotides during polymerization [12,107]. Taken together, XNA aptamers could find widespread use as molecular recognition elements in diagnostic and therapeutic applications, especially where high biological stability is required. These novel technologies could help drive new innovations in synthetic biology as well as molecular medicine.

## 9. Emerging Opportunities in a Pandemic Era

Early and precise detection of the virus is important for effective diagnosis and treatment of diseases, especially for those with a public health emergency and international concern. The unprecedented spread of SARS-CoV-2 and the dire pandemic situation of COVID-19 now demands improvement of current diagnostic methods, as well as the development of vaccines and therapeutics [108]. Fast evolving situations including novel mutants of SARS-CoV-2 require shorter development time and flexibility for wider applications. Aptamers, such as chemical antibodies or synthetic antibodies, are suited for this kind of application (Figure 2). So far, aptamers that target the whole virus or surface antigens have been developed for viruses such as hepatitis B and C viruses [41,109], human papilloma virus [110], HIV [111], influenza [60,112], SARS [14,37], Ebola [113], dengue [114], herpes simplex virus [115], and West Nile virus [116]. In addition, various testing kits and assays such as ELISA-based immunoassays, real-time reverse transcriptase PCR, and point-of-care (POC) have been implemented to detect the virus; however, these approaches have inherent limitations such as inadequate specificity and long turnaround times for the test results. Therefore, aptamers could offer an alternative to generate high affinity ligands for monitoring of relevant SARS-COV-2 and COVID-19 biomarkers [37]. A rapid, sensitive, and specific diagnostic platform would aid in stopping the spread of the virus. Moreover, in silico methods and artificial intelligence can be used to identify a specific aptamer for improved aptasensor technologies against the SARS-CoV-2, consequently speeding up the design and development of point-of-care testing for the virus. While RT-PCR is currently the most widely used test formats for COVID-19, the speed and convenience of lateral flow test (LFT) based on an aptamer would increasingly find its application in a pandemic era.

**Figure 3:**
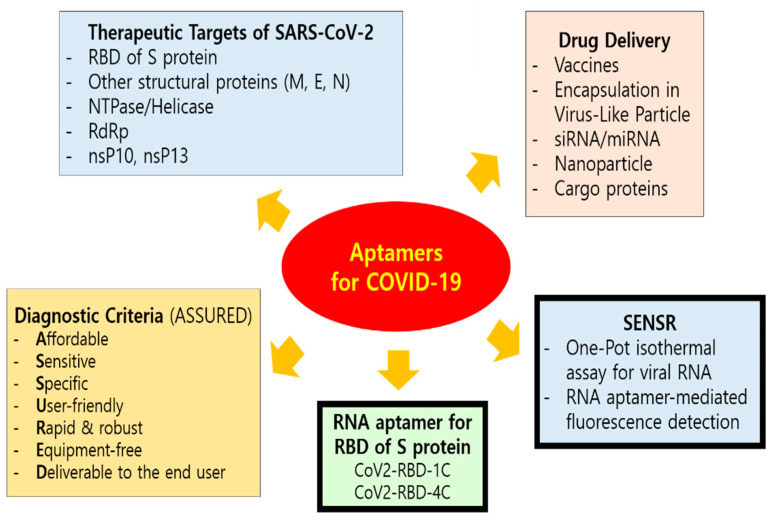
Current efforts of aptamer application for COVID-19. Aptamers can be engineered for the development of effective and affordable diagnostics, therapeutics, and prophylactic vaccines for SARS-CoV-2. Rapid and accurate detection methods, as well as smart vaccine and drug designs with SARS-CoV-2 targeting capabilities, are critically needed. The ASSURED (affordable, sensitive, specific, user-friendly, rapid and robust, equipment-free, and deliverable to the end user) diagnostic criteria is recommended by WHO. The mode of entry for SARS-CoV-2 into host cells occurs through clathrin-mediated endocytosis which depends on the binding interaction between the receptor-binding domain (RBD, Arg319-Phe541) at the C-terminus of spike proteins (S) of SARS-CoV-2 and the host cellular receptors, ACE2, along with S proteins priming by host cell transmembrane serine protease 2 (TMPRSS2). Coronaviruses have four structural proteins, i.e., spike (S), envelope (E), membrane (M) and nucleocapsid (N) that facilitate interactions with the host cell and activities within the host after internalization [108]. On the basis of the sequence similarity between SARS-CoV and SARS-CoV-2, modifications in sequence and structure of SARS-CoV aptamers may produce specific aptamers for SARS-CoV-2 proteins, including N [118].

In 2020, high-binding-affinity aptamers targeting the RBD (receptor-binding domain) of the SARS-CoV-2 Spike glycoprotein (Figure 3) were identified using an ACE2 competition-based aptamer selection strategy and a machine learning screening algorithm [117]. The Kd values of the optimized aptamers (CoV2-RBD-1C and CoV2-RBD-4C) against the RBD were 5.8 nM and 19.9 nM, respectively. According to molecular docking and molecular dynamic simulations and competition experiments between the aptamers and ACE2, the two aptamers were suggested to have partially identical binding sites at ACE2 on the SARS-CoV-2 RBD. These aptamers could be useful for recognition of SARS-CoV-2 and could facilitate the diagnosis and treatment of SARS-CoV-2 while providing a valuable tool for elucidating the mechanisms behind the coronavirus entry into cells.

In addition, a highly sensitive and specific one-pot isothermal assay (SENSR) for the fluorescence-based detection of SARS-CoV-2 RNA in 30–50 minutes has been developed [119]. The assay involves the hybridization and ligation of promoter and reporter DNA probes, followed by the transcription and amplification of an RNA aptamer that binds to a fluorescent dye. The RNA aptamer is transcribed by the T7 RNA polymerase from the ligation product (a promoter DNA probe plus a reporter DNA probe) that hybridize with the target single-stranded RNA sequence. Ligation of the two DNA probes is mediated by the SplintR ligase which is a chlorella virus DNA ligase. SplintR ligase is 100× faster than either T4 DNA ligase or T4 RNA ligase 2 for RNA splinted DNA ligation [120]. The achieved positive and negative predictive values were 95% and 100%, respectively, when tested with 40 nasopharyngeal samples with half of them positive for SARS-CoV-2. With a limit of detection at 0.1 attomolar RNA concentration, the assay can rapidly detect a range of viral and bacterial RNAs and should be amenable to paper and lateral-flow formats [119]. 

Recently, new and highly effective fluorogenic aptamers called Mango aptamers were developed using a competition-based, ultrahigh-throughput fluorescent screening approach that takes advantage of microfluidic-assisted in vitro compartmentalization [121,122,123]. The RNA Mango technology is based on the specific binding of the RNA Mango Aptamer and a thiazole orange (TO) derivative, TO1-Biotin, which is a bi-functional dye. The dye only fluoresces when bound to the Mango aptamer. The main features of this technology are the tight binding between the dye and aptamer and the strong enhancement of the dye’s fluorescence (up to 1100-fold) when bound to the Mango aptamer. The full biological compatibility of Mango aptamers is still not completely explored; however, their small size relative to other fluorogenic RNA aptamers and their ability to fold correctly into monomers at physiological temperatures, combined with their unusual ability to withstand formaldehyde fixation [122], all promise to be very useful for live-cell imaging of small non-coding RNAs or tracking the behavior of tagged molecules including the RNA viruses such as SARS-CoV-2.

Next generation aptamers have seen improvements in their designs. Allosteric regulation for the transient loading and release of small organic molecules has also been investigated [124]. Two model DNA-based aptamers that bind ATP and cocaine through a target-induced conformational change have been re-engineered so that their affinity towards their specific target is controlled by a DNA sequence acting as an allosteric inhibitor. The use of an enzyme that specifically cleaves the inhibitor only when it is bound to the aptamer generates a transient allosteric control that leads to the release of ATP or cocaine from the aptamers. This approach confirms that the programmability and predictability of nucleic acids make synthetic DNA/RNA the perfect candidate material to re-engineer synthetic receptors that can undergo chemical fuel-triggered release of small-molecule cargoes, and therefore rationally design non-equilibrium systems [124]. From another perspective, drug-antidote pairs could be rationally designed by taking advantage of properties inherent in nucleic acids to make antidote-controlled aptamers [125]. Since the selection of the first thrombin-binding aptamer in 1992, the use of nucleic acid aptamers to target specific coagulation factors has emerged as a valuable approach for generating novel anticoagulant and procoagulant therapeutics. A complementary oligonucleotide (antidote) that hybridizes with the aptamer provides a unique opportunity to control the duration of the therapeutic action [125,126].

## 10. Conclusions

Recent advances in nucleic acid engineering and theragnostics are paving the way for nucleotide aptamers. These small nucleic acid ligands that are composed of RNA or single-stranded DNA oligonucleotides bind to their target with high specificity and affinity. Over the last three decades, the field of aptamers has grown from its initial focus targeting small molecules and soluble proteins to include cell surface targets and organs [8,9]. Similar to antibodies, aptamers interact with their targets by recognizing a specific three-dimensional structure. The ability of aptamers to mimic antibodies as chemical antibodies has stimulated many biomedical applications, including therapeutics, diagnostics, and drug delivery. The unique characteristics and benefits of aptamers are promising for the development of novel therapeutics and diagnostics for viral infections such as COVID-19. RNA aptamers such as Mango have recently emerged as a powerful background-free technology for live-cell RNA imaging due to their fluorogenic properties upon ligand binding [123]. XNA aptamers and aptasensors are also driving new innovations, which should aid in the development of reliable point-of-care test for SARS-CoV-2. Therapeutic and prophylactic applications of aptamers are also emerging. These developments provide a glimpse into the future of possible applications for aptamers. However, multiple challenges such as optimization of selectivity, stability, delivery, and long-term safety, as well as reproducibility have to be addressed for nucleic acid aptamers to become a successful drug and research tool [127]. Detailed mechanisms on how aptamers bind to their targets must also be elucidated. Making reliable aptamers is a pressing issue for successful clinical translation. Proper and thorough testing of the candidate aptamer with every appropriate control is required. In conclusion, while there are still some gaps in developing aptamers for clinical applications, aptamers still have enormous potential that will restore initial promises.

## Figures and Tables

**Table 1:** Comparison of an antibody and an aptamer.

Characteristics	Antibody	Aptamer
Target site is determined by Toxic or poorly immunogenic targets Batch-to-batch variation Scaffold Production Storage Shelf life Supply Pharmacokinetic parameters Chemical modifications Controllable binding and release condition Size Denatured by heat Immunogenicity Isolation process Screening time and cost Cross-reactive compounds isolation	Immune system Yes Yes Monoclonal antibody Hybridoma culture Hybridoma Limited Liquid, cold chain difficult to modify difficult No Large Yes Significant In vivo Time-consuming and expensive No method available	Investigator No No No fixed scaffold Solid phase synthesis Sequence ID Long Dry, room temperature can be changed on demand easy Yes Small No Little to None In vitro Iterative rounds against known target limit screening processes Toggle strategy

**Table 2:** Aptamers in clinical trial as of 29 January 2021.

Aptamer	Type	Target	Indications	Status
Macugen (Pegaptanib)		VEGF	AMD (Age-related macular degeneration)	Approved *
	RNA	VEGF	PDR (Proliferative diabetic retinopathy)	Phase 1, completed
		VEGF	DME (Diabetic macular edema)	Phase 2, completed *
EYE001		VEGF	Macular degeneration	Phase 2, Completed
	RNA		Choroidal neovascularization	Phase 3, completed
		VEGF	Von Hippel–Lindau disease	Phase 1, completed
Fovista (E10030)	DNA	PDGF-BB	AMD (Age-related macular degeneration)	Phase 2, terminated *
		PDGF	AMD (Age-related macular degeneration)	Phase 2, completed *
AS1411	DNA	Nucleolin	AML (Acute myeloid leukemia)	Phase 2, terminated
ARC1779	DNA	vWF	von Willebrand disease	Phase 2, withdrawn
		vWF	Purpura, thrombotic thrombocytopenic von Willebrand disease type-2b	Phase 2, completed
ARC19499	RNA	TFPI	Hemophilia	Phase 1, terminated
ARC1905 (Zimura)	RNA	C5	AMD (Age-related macular degeneration)	Phase 1, completed *
		C5	Geographic atrophy	Phase 2, completed
			Macular degeneration	Phase 3, completed
		C5	IPCV (idiopathic polypoidal choroidal vasculopathy)	Phase 2, completed
REG1	RNA	Factor IX	Coronary artery disease	Phase 3, terminated *^,^†
Nox-E36	RNA	MCP-1(CCL2)	Chronic inflammatory diseases Type 2 diabetes mellitus Systemic lupus erythematosus	Phase 1, completed
Nox-A12	RNA	SDF-1(CXCL12)	Autologous stem cell transplantation	Phase 1, completed
Nox-H94	RNA	Hepcidin	Anemia End stage renal disease	Phase 1, completed Phase 2, completed
BT200	RNA	vWF, Factor VIII	von Willebrand disease Hemophilia A	Phase 2, recruiting
68Ga-Sgc8	DNA	PTK7 (CCK4)	Colorectal cancer	Phase 1, unknown
NU172	DNA	Thrombin	Heart disease	Phase 2, unknown

* When there are multiple cases with the same drugs and indications, the most advanced trial or the one with known status was shown. PDR, proliferative diabetic retinopathy; DME, diabetic macular edema; † clinical hold.BT200 is a PEGylated aptamer that binds to the A1 domain of human von Willebrand factor (VWF). NOX-A12 targets CXCL12 (C-X-C chemokine ligand 12), a key chemokine (signaling) protein. NU172, a 26-mer oligonucleotide able to bind exosite I of human thrombin and inhibit its activity, was the first aptamer to reach Phase II clinical studies as an anticoagulant in heart disease treatments.

**Table 3:** Selected examples of aptamer immobilization on surfaces.

Analytical Methods	Aptamer Conjugation/Spacer	Architecture	Reference
Surface plasmon resonance	Biotin	Streptavidin-sensor chip	[77]
Surface plasmon resonance	None	Graphene oxide-coated gold chip	[78]
Electrochemical	Biotin/T_5_	Streptavidin-gold electrode	[79]
Electrochemical impedance spectroscopy (EIS)	NH_2_	Poly(amidoamine) dendrimers immobilized on gold electrode	[80]
Lateral flow assay	Biotin	Streptavidin-nitrocellulose membrane	[81]
Capture and colorimetric detection	None	Gold nanoparticles	[82]
Capture and detection by fluorescence	Biotin	Streptavidin-silica nanoparticles	[83]

Requirements of the desired analytical methods determine the choice of substrate and chemistry for aptamer immobilizataion.

**Table 4:** Limitations of 1st generation aptamers and their solutions.

Limitations	Solutions
Rapid renal excretion	Conjugation of high M.W. PEG or Cholesterol
Nuclease-mediated degradation	Chemical modification at 2’ or 3’ terminus, mirror-image aptamer (spiegelmer)
Susceptibility to hydrolysis	Chemically substituted nucleotide analogs during SELEX
Toxicity	Minimal toxicity, more rigorous studies are needed
Intracellular targets	Intramer, intracellular expression, receptor-mediated endocytosis
Absence of control	Antidote (a complementary oligonucleotide)
Loss of targeting	Modulation of immune responses
Production yield	Incorporation of protective groups, large scale manufacturing, solid phase synthesis, flow-through technology

## Data Availability

Not applicable.
